# Quantitative MRI Harmonization to Maximize Clinical Impact: The RIN–Neuroimaging Network

**DOI:** 10.3389/fneur.2022.855125

**Published:** 2022-04-14

**Authors:** Anna Nigri, Stefania Ferraro, Claudia A. M. Gandini Wheeler-Kingshott, Michela Tosetti, Alberto Redolfi, Gianluigi Forloni, Egidio D'Angelo, Domenico Aquino, Laura Biagi, Paolo Bosco, Irene Carne, Silvia De Francesco, Greta Demichelis, Ruben Gianeri, Maria Marcella Lagana, Edoardo Micotti, Antonio Napolitano, Fulvia Palesi, Alice Pirastru, Giovanni Savini, Elisa Alberici, Carmelo Amato, Filippo Arrigoni, Francesca Baglio, Marco Bozzali, Antonella Castellano, Carlo Cavaliere, Valeria Elisa Contarino, Giulio Ferrazzi, Simona Gaudino, Silvia Marino, Vittorio Manzo, Luigi Pavone, Letterio S. Politi, Luca Roccatagliata, Elisa Rognone, Andrea Rossi, Caterina Tonon, Raffaele Lodi, Fabrizio Tagliavini, Maria Grazia Bruzzone, The RIN–Neuroimaging

**Affiliations:** ^1^U.O. Neuroradiologia, Fondazione IRCCS Istituto Neurologico Carlo Besta, Milan, Italy; ^2^MOE Key Laboratory for Neuroinformation, School of Life Science and Technology, University of Electronic Science and Technology of China, Chengdu, China; ^3^Unità di Neuroradiologia, IRCCS Mondino Foundation, Pavia, Italy; ^4^NMR Research Unit, Department of Neuroinflammation, Queen Square MS Centre, UCL Queen Square Institute of Neurology, Faculty of Brain Sciences, University College London, London, United Kingdom; ^5^Department of Brain and Behavioral Sciences, University of Pavia, Pavia, Italy; ^6^Medical Physics and MR Lab, Fondazione IRCCS Stella Maris, Pisa, Italy; ^7^Laboratory of Neuroinformatics, IRCCS Istituto Centro San Giovanni di Dio Fatebenefratelli, Brescia, Italy; ^8^Laboratory of Biology of Neurodegenerative Disorders, Istituto di Ricerche Farmacologiche Mario Negri IRCCS, Milan, Italy; ^9^Neuroradiology Unit, IRCCS Istituti Clinici Scientifici Maugeri, Pavia, Italy; ^10^IRCCS Fondazione Don Carlo Gnocchi Onlus, Milan, Italy; ^11^Medical Physics, IRCCS Istituto Ospedale Pediatrico Bambino Gesù, Rome, Italy; ^12^Neuroradiology Unit, IRCCS Humanitas Research Hospital, Milan, Italy; ^13^Unit of Neuroradiology, Oasi Research Institute-IRCCS, Troina, Italy; ^14^Neuroimaging Unit, Scientific Institute, IRCCS E. Medea, Bosisio Parini, Italy; ^15^Neuroimaging Laboratory, Santa Lucia Foundation, IRCCS, Rome, Italy; ^16^Neuroradiologia, IRCCS Ospedale San Raffaele, Milan, Italy; ^17^IRCCS Synlab SDN, Naples, Italy; ^18^Unità di Neuroradiologia, Fondazione IRCCS Ca' Granda Ospedale Maggiore Policlinico, Milan, Italy; ^19^IRCCS San Camillo Hospital, Venice, Italy; ^20^Istituto di Radiologia, UOC Radiologia e Neuroradiologia, IRCCS Fondazione Policlinico Universitario Agostino Gemelli, Rome, Italy; ^21^IRCCS Centro Neurolesi “Bonino-Pulejo”, Messina, Italy; ^22^Department of Radiology, Istituto Auxologico Italiano, IRCCS, Milan, Italy; ^23^IRCCS Neuromed, Pozzilli, Italy; ^24^Department of Biomedical Sciences, Humanitas University, Milan, Italy; ^25^Neuroradiologia IRCCS Ospedale Policlinico San Martino, Genoa, Italy; ^26^Dipartimento di Scienze della Salute Università di Genova, Genoa, Italy; ^27^UO Neuroradiologia, IRCCS Istituto Giannina Gaslini, Genoa, Italy; ^28^Functional and Molecular Neuroimaging Unit, IRCCS Istituto delle Scienze Neurologiche di Bologna, Bologna, Italy; ^29^Scientific Direction, Fondazione IRCCS Istituto Neurologico Carlo Besta, Milan, Italy

**Keywords:** harmonization, multisite, quantitative MRI, QSM, diffusion MRI, fMRI, neuroimaging

## Abstract

Neuroimaging studies often lack reproducibility, one of the cardinal features of the scientific method. Multisite collaboration initiatives increase sample size and limit methodological flexibility, therefore providing the foundation for increased statistical power and generalizable results. However, multisite collaborative initiatives are inherently limited by hardware, software, and pulse and sequence design heterogeneities of both clinical and preclinical MRI scanners and the lack of benchmark for acquisition protocols, data analysis, and data sharing. We present the overarching vision that yielded to the constitution of *RIN-Neuroimaging Network*, a national consortium dedicated to identifying disease and subject-specific *in-vivo* neuroimaging biomarkers of diverse neurological and neuropsychiatric conditions. This ambitious goal needs efforts toward increasing the diagnostic and prognostic power of advanced MRI data. To this aim, 23 Italian Scientific Institutes of Hospitalization and Care (IRCCS), with technological and clinical specialization in the neurological and neuroimaging field, have gathered together. Each IRCCS is equipped with high- or ultra-high field MRI scanners (i.e., ≥3T) for clinical or preclinical research or has established expertise in MRI data analysis and infrastructure. The actions of this Network were defined across several work packages (WP). A clinical work package (WP1) defined the guidelines for a minimum standard clinical qualitative MRI assessment for the main neurological diseases. Two neuroimaging technical work packages (WP2 and WP3, for clinical and preclinical scanners) established *Standard Operative Procedures* for quality controls on phantoms as well as advanced harmonized quantitative MRI protocols for studying the brain of healthy human participants and wild type mice. Under FAIR principles, a web-based e-infrastructure to store and share data across sites was also implemented (WP4). Finally, the RIN translated all these efforts into a large-scale multimodal data collection in patients and animal models with dementia (i.e., case study). The *RIN-Neuroimaging Network* can maximize the impact of public investments in research and clinical practice acquiring data across institutes and pathologies with high-quality and highly-consistent acquisition protocols, optimizing the analysis pipeline and data sharing procedures.

## Introduction

The identification of early and accurate *in vivo* non-invasive biological markers-“a characteristic that is objectively measured and evaluated as an indicator of normal biological processes, pathogenic processes, or pharmacologic responses to a therapeutic intervention” (1)-in brain tissue is a crucial endpoint in neuroimaging research (2–6).

Multisite collaboration initiatives allow accruing large-scale quantitative magnetic resonance imaging (qMRI) data paving the way to overcome the current replication crisis in neuroimaging science (7–10) and to data-driven analysis methods, through machine/deep-learning techniques (11, 12), fundamental tools in the identification of reliable neuroimaging biomarkers (13). These initiatives are also important opportunities for sharing technical and scientific knowledge, new ideas, and available resources. Initiatives such as the Alzheimer's Disease Neuroimaging Initiative (ADNI, http://adni.loni.usc.edu/), ESR/EIBALL (https://www.myesr.org/research/european-imaging-biomarkers-alliance-eiball), Quantitative Imaging Biomarkers Alliance (QIBA) (https://www.rsna.org/research/quantitative-imaging-biomarkers-alliance) (14), or Biomedical Informatics Research Network (BIRN) (15, 16) are successful examples of this intention.

Defining qMRI sequences, and standardized procedures for their quality control and for data analysis and sharing within a network of research institutes providing ongoing support according to their specific expertise is a much-needed “conditio sine qua non” if the ultimate aim is to promote the translation of such methods into the clinical context of research hospitals. Within such a network, with expertise that bridges across disciplines (radiology, neurology, physics, computer science, statistics), protocols and standard operating procedures (SOPs) can meet the standards for benchmarking against an MRI technology in constant evolution, with scanners that present a vast heterogeneity in terms of their characteristics (e.g., manufacturer, gradient system, transmitter/receiver coils, sequences, software version).

A recent survey on the current state of neuroimaging biomarker harmonization unmet needs identified different high-level barriers. Amongst them, the lack of guidelines or regulations for the harmonization of data acquisition and analyses, the cost underestimation for infrastructures, the need for qualified and experienced personnel, remain the most important challenging issues (17).

Calls to action to overcome those barriers should include the harmonization of quality controls (QC) procedures to evaluate the performance of the scanners concerning reference values (6, 18, 19) and the harmonization of multivendor state-of-the-art acquisition protocols to guarantee the repeatability and the reproducibility of qMRI measures inter-/intra-scanner (20–24), as well as the setting up of IT infrastructures suitable for data exchange and sharing (25, 26). A network of highly trained personnel, well-integrated with the clinical teams, supporting the implementation across sites, is what can make the real difference in terms of successful clinical impact.

If these issues are well identified and talked about in clinical neuroimaging research settings, they are hardly mentioned in preclinical imaging research (27). In the last few years, the reproducibility of results in preclinical research has shown itself as an important basis for successful clinical trials (27). Unfortunately, only a few multisite studies have been carried on so far. Preclinical multisite harmonization has been limited to a few centers (i.e., 2–3 sites) with the same experimental setup (28, 29) or to the application of shared pipeline analysis to data obtained with different experimental setups (30). Therefore, a multisite coordination that brings together both clinical and technical expertise is a fundamental step for structured large-scale data collection. Moreover, it would be appropriate for this coordination to offer ongoing support for internal and external users. This is a priority not only within clinical research but also for preclinical research and translational studies (31, 32).

### The Neuroscience and Neurorehabilitation Network (RIN): RIN–Neuroimaging Network

Under the increasing pressure of the burden and cost of neurological and neuropsychiatric diseases (33, 34), the Italian Ministry of Health in 2017 founded the *Neuroscience and Neurorehabilitation Network (RIN)* the Italian largest research network in the neuroscience field. *RIN* drives to collaboration Scientific Institutes of Hospitalization and Care (IRCCSs). One of the fundamental branches of RIN is the *RIN–Neuroimaging Network* (https://www.reteneuroscienze.it/en/progetti/neuroimaging/), which main ambitious goal is to identify the disease and subject-specific *in-vivo* neuroimaging biomarkers of diverse neurological and neuropsychiatric conditions. This ambitious goal needs efforts toward increasing the diagnostic and prognostic power of advanced MRI data, requiring, as the first essential step, to specify guidelines and SOPs, to be readily adopted by IRCCS, for data acquisitions, processing, and sharing of disease-specific MRI protocols.

### Main Goals

To achieve the main purpose of the *RIN–Neuroimaging Network, four* initial operational goals have been identified:

Goal 1: definition of shared MRI protocols for the main neurological diseases;

Goal 2: quality controls (QC) on *ad hoc* phantoms;

Goal 3: harmonization of advanced MRI protocol in clinical and preclinical research;

Goal 4: setting up communication infrastructure and data management.

## Methods and Analysis

### Design

The initial core of the *RIN–Neuroimaging Network*, constituted by neuroradiologists, physicists, and engineers of participating IRCCSs, was formalized according to the following criteria:

- To be active in neurology research;
- To have a high field scanner (3T) or ultra-high-field scanner (7T) for clinical or preclinical research or well-known expertise in MRI data analysis and infrastructure management.

**Figure 1:**
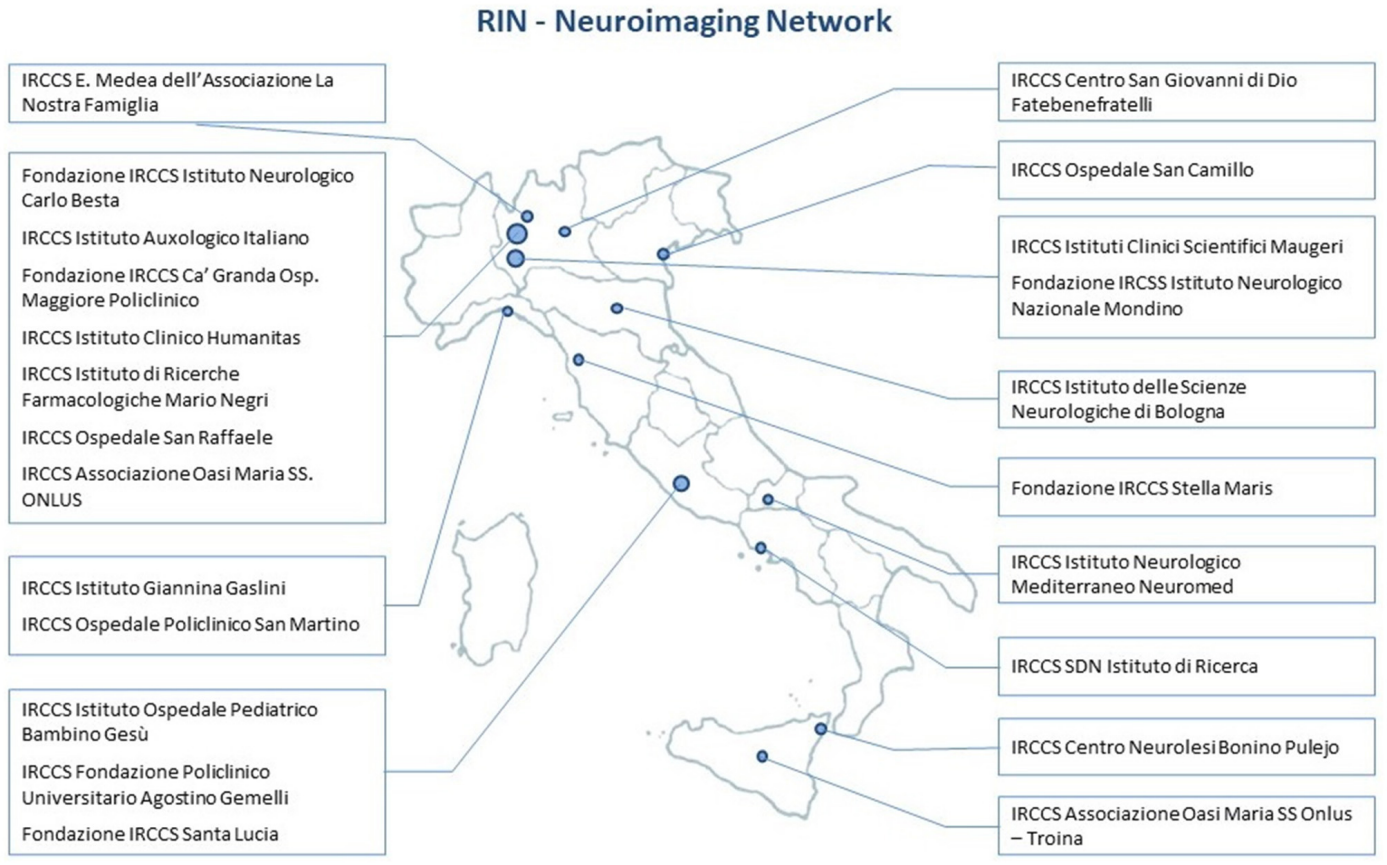
Listing and geographical distribution of the 23 Italian sites of the *RIN–Neuroimaging Network*.

All IRCCSs were contacted. Before their formal involvement in *RIN–Neuroimaging Network*, the technological and clinical specialization of each IRCCS was verified through a detailed survey (Figure 1, Table 1).

**Table 1:** Scanner and equipment for each site of *RIN–Neuroimaging Network*.

**n**.	**Code**	**Site**	**Clinical MRI scanner**				**Preclinical MRI scanner**				**IT Infrastructure**
			**Field (T)**	**Vendor**	**Model**	**Head coil**	**Field (T)**	**Vendor**	**Scanner**	**Head coil**	
1	EM	IRCCS E. Medea dell'Associazione La Nostra Famiglia	3	Philips	Achieva dStream	32 ch	-	-	-	-	-
2	BP	IRCCS Centro Neurolesi Bonino Pulejo	3	Philips	Achieva dStream	32 ch	-	-	-	-	-
3	CB	Fondazione IRCCS Istituto Neurologico Carlo Besta	3	Philips	Achieva → Achieva dStream	32 ch	7	Bruker	Biospec 70/20	quadrature	-
4	SL	Fondazione IRCCS Santa Lucia	3	Philips	Achieva → Achieva dStream	32 ch	-	-	-	-	-
5	CG	Fondazione IRCCS Ca' Granda Osp. Maggiore Policlinico	3	Philips	Achieva dStream	32 ch	-	-	-	-	-
6	SC	IRCCS Ospedale San Camillo	3	Philips	Ingenia	32 ch	-	-	-	-	-
7	GG	IRCCS Istituto Giannina Gaslini	3	Philips	Ingenia	32 ch	-	-	-	-	-
8	SR	IRCCS Ospedale San Raffaele	3	Philips	Ingenia	32 ch	7	Bruker	Biospec 70/30	quadrature	-
9	Hu	IRCCS Istituto Clinico Humanitas	3	Siemens	Verio → SkyraFit	8 ch → 64 ch	-	-	-	-	-
10	CM	Fondazione IRCSS Istituto Neurologico Nazionale Mondino	3	Siemens	Skyra	32 ch	-	-	-	-	-
11	BG	IRCCS Istituto Ospedale Pediatrico Bambino Gesù	3	Siemens	Skyra	32 ch	-	-	-	-	-
12	SN	IRCCS Istituto delle Scienze Neurologiche di Bologna	3	Siemens	Skyra	64 ch	-	-	-	-	-
13	DG	IRCCS Fondazione Don Carlo Gnocchi Onlus	3	Siemens	Prisma	64 ch	-	-	-	-	-
14	SM	IRCCS Ospedale Policlinico San Martino	3	Siemens	Prisma	64 ch	-	-	-	-	-
15	SDN	IRCCS SDN Istituto di Ricerca	3	Siemens	Siemens Biograph_mMR	12 ch	-	Bruker	Biospec 94/20	4 ch	-
16	NE	IRCCS Istituto Neurologico Mediterraneo Neuromed	3	GE	GE Signa HDxt	8 ch	7	Bruker	Pharmascan 70/16	quadrature	-
17	MA	IRCCS Istituti Clinici Scientifici Maugeri	3	GE	GE Discovery MR750	16 ch	-	-	-	-	-
18	AU	IRCCS Istituto Auxologico Italiano	3	GE	GE Discovery MR750	32 ch	-	-	-	-	-
19	MN	IRCCS Istituto di Ricerche Farmacologiche Mario Negri	-		-	-	7	Bruker	Biospec 70/30	quadrature	-
20	ST	Fondazione IRCCS Stella Maris	7 *	GE	Discovery MR950 → SIGNA7T	Tx 2ch/ Rx 32ch	-	-	-	-	Arianna (https://arianna.pi.infn.it/it)
				Acquiring GE 3T PREMIER clinical scanner							
21	FBF	IRCCS Centro San Giovanni di Dio Fatebenefratelli	-		-	-	-	-	-	-	NeuGRID (https://www.neugrid2.eu/)
22	OM	IRCCS Associazione Oasi Maria SS Onlus – Troina (EN)			Acquiring 3T clinical scanner		-	-	-	-	-
23	AG	IRCCS Fondazione Policlinico Universitario Agostino Gemelli			Acquiring 3T clinical scanner		-	-	-	-	-

The symbol “ → ” in the “model” column indicates the scanners, whose hardware was updated since the beginning of the project. (^*^) The ultra-high-field clinical 7T scanner was used to test the feasibility and the improvement of quantitative MRI protocol for future implementation on this system. IRCCS, Scientific Institutes of Hospitalization and Care; T, tesla; GE, General Electric; ch, channels.

**Figure 2:**
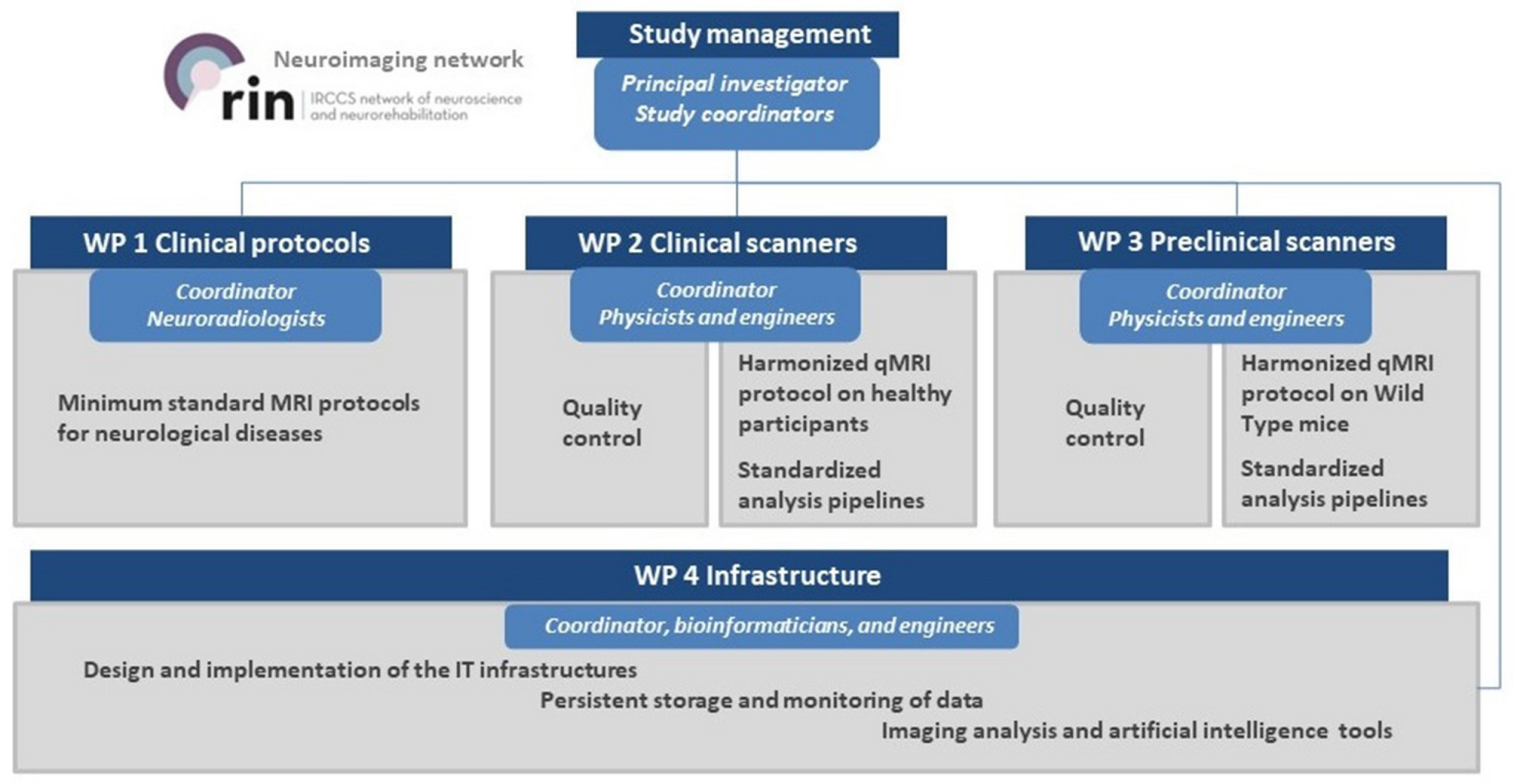
Project flow chart and work packages organization.

The operational goals of the *RIN–Neuroimaging Network* were pursued through the following work packages (WP): *WP1 clinical protocols, WP2 clinical scanners, WP3 preclinical scanner, WP4 infrastructure*. Each IRCCS organized its participating staff into working groups (see Figure 2). Every working group reported their activity and WP progress every 6 months during a consensus meeting.

### Interventional Methods

The Network planning was organized into several parallel working phases.

*WP1 was* conducted by experienced radiologists and was responsible for identifying the biophysical characteristics of an advanced qMRI protocol to be harmonized across the different sites in *WP2*. Radiologists *WP1* focused on identifying the main pathology of interest for the largest number of sites to define guidelines for the acquisition of a minimum standard clinical MRI protocol. Finally, *WP1* selected a case study of the pathology of interest for the largest number of sites to apply the advanced harmonized qMRI protocol using both clinical (*WP2*) and preclinical scanners (*WP3*).

*WP2*, delegated to experienced physicists and engineers, was organized into the following project tasks: (i) survey of MRI scanner and related equipment (e.g., vendor, gradient system, transmitter/receiver coils, software release, year of installation); (ii) selection of QC phantoms and definition of SOP to test scanner's performance (i.e., intra- and inter-scanner repeatability and reproducibility); (iii) set up and development of a harmonized qMRI protocol, described in an SOP and tested on healthy participants of similar age and gender; (iv) delineation of reproducibility standards for each qMRI metric as a possible biomarker across sites.

*WP3*, delegated to experienced physicists and engineers, was divided into the following project tasks: (i) use of standard Bruker and n-Tridecane phantoms (35) for the evaluation of preclinical scanner's performance; (ii) identification of a harmonized qMRI protocol on mice; (iii) delineation of reproducibility standards for each qMRI metric across sites.

*WP4*, carried out by experienced bioinformaticians and engineers, dealt with the (i) design and implementation of the IT infrastructures for data management and communication, according to the Findable, Accessible, Interoperable, and Reusable (FAIR) principles (36); (ii) implementation of analysis pipelines and data analytics using artificial intelligence and machine learning.

### Data Analyses

#### WP1: Clinical Protocols

Twelve neurological diagnostic classes of interest were identified: dementia (Alzheimer's disease, frontotemporal dementia, vascular dementia), epilepsy, demyelinating diseases, motor neuron diseases, spinal cord pathologies, Parkinson's disease and parkinsonism, brain tumors, disorders of consciousness, rare diseases, pediatric diseases, headaches, and cerebrovascular diseases.

**Figure 3:**
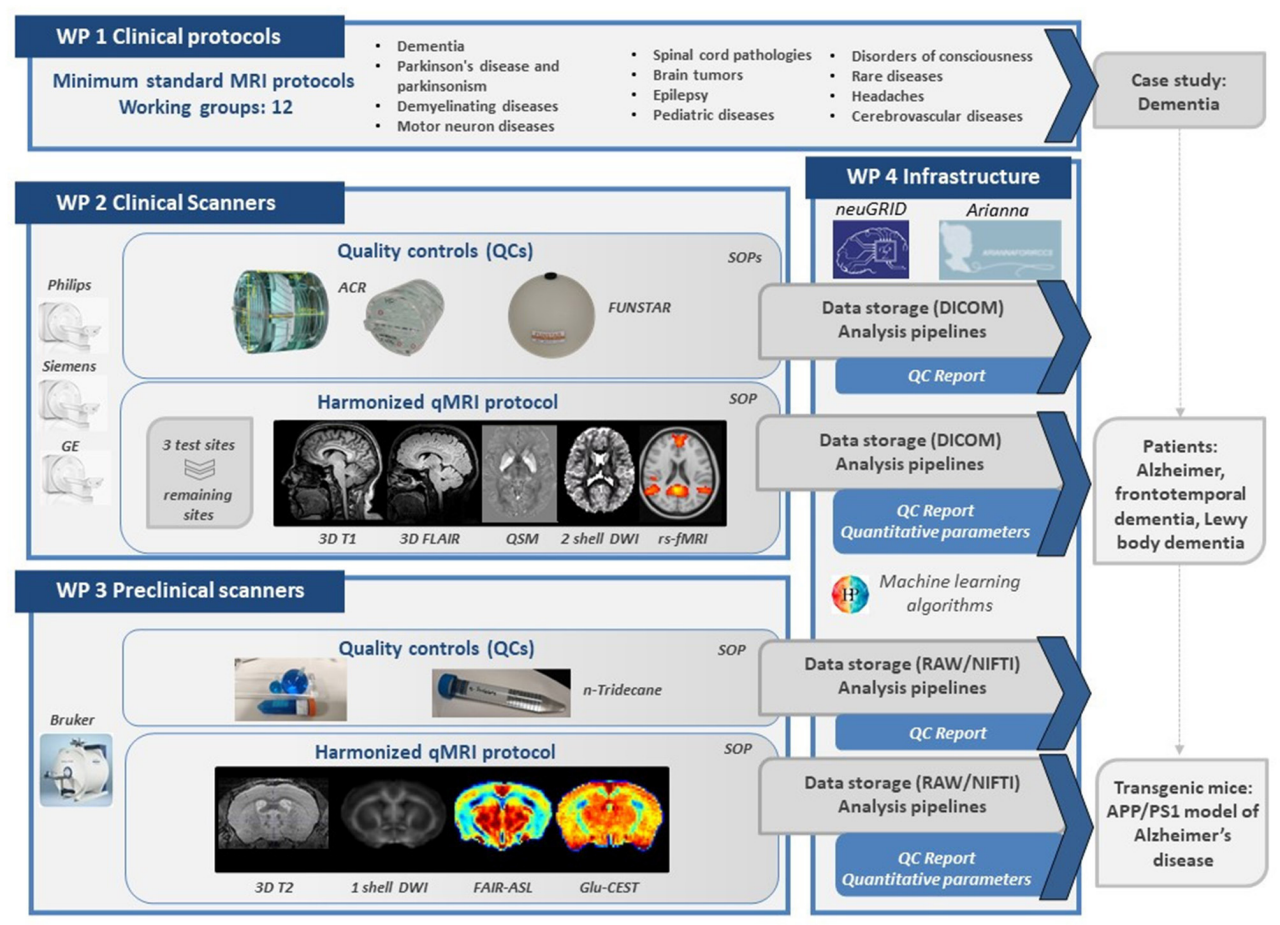
Schematic representation of the main results, roadmap, and integration between the 4 project WPs. WP, work packages; SOP, standard operating procedure; QSM, quantitative susceptibility mapping; DWI, diffusion-weighted imaging; rs-fMRI, resting-state functional magnetic resonance imaging; FAIR-ASL, flow-sensitive alternating inversion recovery arterial spin labeling; Clu-CEST, glutamate chemical exchange saturation transfer.

For each of these neurological diagnostic classes, neuroradiologists have established *guidelines for the acquisition of a minimum standard clinical MRI protocol* that includes the sequences required for radiological evaluation, as well as relevant geometry imaging parameters (i.e., slice orientation, phase encoding, the range for the field of view, voxel size, slice thickness). These guidelines were shared with the Italian Association of Neuroradiology for a wider application and diffusion to various centers at the national level. Neuroradiologists and researchers delineated MRI sequences for an advanced qMRI protocol, which includes techniques for both clinical, structural, and functional assessment of the brain according to the state of the art in the neuroimaging field. The working group identified its case study in dementia so that it can initiate the translation of all harmonization efforts into large-scale multimodal data collection for patients and animal models (Figure 3).

#### WP2: Clinical Scanners

The 3T scanner survey showed that within the Network there was a prevalence of Philips vendor (8 scanners), compared to General Electric (GE) (3 scanners) and Siemens (7 scanners) vendors. Moreover, heterogeneity in scanner configurations was found, particularly in the gradient system and the transmitter/receiver head coils. A head coil with 32 or fewer channels was available in 15 sites, while the latest technology with a 64 channels head coil was available in 3 centers. The size of the head coils affected the choice of phantoms for the QCs. QC phantom scans were conducted approximately monthly for 2 to 4 years. Through QC, we assessed the reproducibility over time among scanners, using published reference performance indices even when significant hardware and software upgrades were performed intra-site in response to the progress of technology.

For the evaluation of geometry and contrast parameters in QC, the American College of Radiology (ACR) phantom was identified and purchased in the large and/or small versions (37): the ACR large phantom fits head coils with 32 channels or less, while the ACR small phantom can fit head coils with 64 channels. For both phantoms, the MRI protocol, including T1- and T2-weighted sequences, was set up starting from the ACR recommendations (37). Additionally, specifications for the in-plane resolution, post-processing filters, and receiver bandwidth were introduced because these parameters are known to affect distortions of the images (38). Thus, *an SOP for QC with ACR phantom* (Figure 3) was established including care of the phantom (e.g., refill), its placement within the head coil, implementation of the MRI protocol, and image acquisition. To implement an automatic QC pipeline, Matlab script available at http://jidisun.wixsite.com/osaqa-project/resources/ was improved (39). This script includes the evaluation of the following quantitative parameters defined in ACR recommendation: geometry distortion, slice thickness accuracy, intensity uniformity, ghosting artifact, and high contrast spatial resolution. A further measure considering the geometric accuracy along the feet-to-head axis was introduced: the ratio between the elliptical areas of the first and the third/fifth slices was still obtained for small/large ACR. Each value derived from QC MRI images was compared with tolerance ranges defined by standard ACR recommendations (37) to define outlier measures.

For the evaluation of the stability of gradient echo-planar imaging (GE-EPI) sequences over time, the FUNSTAR (https://www.goldstandardphantoms.com/products/funstar/) phantom, compatible with all scanner head coils, was chosen. The MRI protocol was set up to use echo-planar sequences with similar parameters affecting gradients' stability and performance. Similar to the ACR phantom, a *SOP for the QC with FUNSTAR phantom* (Figure 3), including placement within the head coil, implementation of the MRI protocol, and image acquisition, was written. Data analysis was performed through a script implemented in Python (https://github.com/mri-group-opbg/stabilitycalc). This analysis estimated the number of parameters that are of interest for neuroimaging functional studies. It computed Signal to Noise Ratio (SNR), Signal to Fluctuation Noise Ratio (SFNR), Percentage Signal Change (PSC), Signal drift, temporal SNR (tSNR) (16, 40). It performed even-odd analysis producing an output to visualize structured noise and spike detection to identify anomalous volumes. Moreover, an additional analysis was implemented in our QC assessment on the FUNSTAR phantom. The Weiskoff analysis that we proposed covered not only a single plane as in the original paper (40), but innovatively all three orthogonal planes as well as the 3D volume in order to estimate the radius of DeCorrelation across all planes. This approach enables us to monitor the effect of noise across the slices and estimate poor slice selection and signal leakage. Values derived from FUNSTAR data were extracted from a central ROI as well as from multiple peripherical ROIs and they were compared with tolerance ranges defined as two standard deviations from the mean value among all the first nine acquisitions from each site, after excluding outliers.

SOPs for QC are available on request at the following link (https://zenodo.org/record/6320896).

After a period of trial and error, an *advanced harmonized qMRI protocol in a clinical setting* was then finalized. The final protocol now includes the following sequences: 3D T1 weighted images (3D T1w), 3D T2 weighted Fluid Attenuated Inversion Recovery (3D T2-FLAIR) images, quantitative susceptibility mapping imaging (QSM), 2-shell diffusion-weighted imaging (DWI), and resting-state functional magnetic resonance imaging (rs-fMRI). 3D T1w and 3D T2-FLAIR images, sagittally oriented, were acquired with the same geometry parameters (e.g., voxel = 1 x 1 x 1 mm^3^, number of slices = 175–180, depending on scanner). For the QSM data, a multi-echo gradient echo sequence (voxel size = 1 × 1 × 1 mm^3^) was defined. Given that different sequence implementations were available across vendors, standardizing the echo-train and echo time (TE) values, without using the scanners in research mode, was quite difficult. So, across all sites, we aimed to achieve a uniform average TE, according to literature standards at the time of implementation (41). For the DWI data, a 2-shell standard single-shot echo-planar imaging sequence (EPI) sequence (voxel size = 2.5 × 2.5 × 2.5 mm^3^, two shells with 30/32-depending on the scanner- isotropically distributed diffusion-weighted directions, diffusion weightings of 1,000 and 2,000 s/mm^2^, 4–7 non-diffusion weighted b = 0 s/mm^2^ images equally distributed among diffusion-weighted images) was implemented. In addition, 3 non-diffusion weighted images with the reversed phase-encoding acquisition were acquired for distortion correction. For the rs-fMRI data, a GE-EPI sequence (voxel size = 3 × 3 × 3 mm^3^, repetition time = 2,400 ms, echo time = 30 ms) was set. GE-EPI inverted blip acquisition was acquired as well. No advanced acceleration parameters were used in any of the protocols, as hardly any of the IRCCS scanners were equipped with options such as simultaneous excitation or compressed sensing as part of their software versions.

The total acquisition time for the qMRI protocol was approximately 40 min.

Thereafter, three test sites were identified: site CB for Philips, site DG for Siemens, site MA for GE. For each sequence of interest, the technical working group defined fundamental geometry and contrast parameters so that each scanner in the Network could implement them. The sequences were implemented and optimized in these 3 test sites. The guidelines on the correct implementation and acquisition of the harmonized qMRI protocol were summarized in an SOP document.

To verify the repeatability and reproducibility of qMRI measures extracted from each sequence, the protocol was acquired in 4 “traveling brains” at each test site. Not only the presence of motion artifacts in both structural and functional sequences but also excessive distortions along the antero-posterior phase encoding direction in DWI and rs-fMRI sequences were evaluated, together with contrast to noise ratio between gray and white matter, signal to noise ratio in different brain tissues and the coefficient of joint variation for the assessment of intensity non-uniformity. After this initial quality check, specific analysis pipelines were implemented based on Freesurfer (https://surfer.nmr.mgh.harvard.edu/) for cortical and subcortical thickness/volume assessment, on STI suite (https://people.eecs.berkeley.edu/~chunlei.liu/software.html) for QSM, on MRTrix/FSL (https://www.mrtrix.org/, https://fsl.fmrib.ox.ac.uk/fsl/fslwiki/FSL) for DWI, and CONN (https://web.conn-toolbox.org/) for rs-fMRI. Several qMRI measures were obtained: cortical and/or subcortical volumes and thickness in regions of interest (ROIs) and corpus callosum shape for 3D T1w; quantitative susceptibility values in subcortical ROIs for QSM; fractional anisotropy, mean diffusivity, mean kurtosis in white matter and gray matter masks for DWI; tSNR in white matter, gray matter, and cerebrospinal fluid masks for rs-fMRI. Similarity within each qMRI measure extracted from the “traveling brains” data was assessed by evaluating intra- and inter-scanner coefficients of variation. In particular, the extracted qMRI measures were considered robust if the coefficients of variation were <10% and no outliers were identified. Therefore, a tolerance range for each qMRI measure was defined as two standard deviations from the mean value calculated across all “traveling brains” data.

After the protocol optimization step in the three test sites, the remaining sites implemented the qMRI protocol following the SOP and/or through direct import of the file/s generated by the test scanners (e.g., examcard, DICOM files). To verify the protocol implementation at each site, the technical working group examined both the quality of the images acquired on a control subject-as outlined for the “traveling brain” -as well as the parameters set through checking automatically specific DICOM tags. Once the protocol was approved, each site proceeded with the acquisition of data using the qMRI protocol in 5 healthy participants (mean age: 29.7 ± 5.0; 32 male/ 45 female). To assess the reliability and robustness of the harmonized protocol across sites, the previously developed analysis pipelines were applied and the qMRI measurements obtained from each subject and each imaging modality were compared with the tolerance range derived from “traveling brains.”

#### WP3: Preclinical Scanners

*WP3* of the *RIN-Neuroimaging Network* defined the first harmonized MRI study-both at the setup and analysis pipeline levels-carried out on a mouse model. The *WP3* produced a *SOP for advanced harmonized qMRI protocol in the preclinical setting*. The survey showed that the 7T preclinical scanners were only from Bruker. Thus, all the QC (SNR, ghosting, intensity stability) were conducted using Bruker standard phantoms. The only exception was for the QC of diffusion measurements for which a homemade phantom filled with n-Tridecane was used to test the stability and reproducibility of diffusion maps (35). The harmonized preclinical protocol includes the following sequences: 3D T2-weighted images (3D T2w), DWI, Flow-sensitive Alternating Inversion Recovey Arterial Spin Labeling (FAIR-ASL), and Glutamate Chemical Exchange Saturation Transfer (Glu-CEST).

3D T2w provide a high resolution 0.1 x 0.1 x 0.1 mm^3^ isotropic anatomical scan. In preclinical imaging, it is imperative to pay attention to the placement of the surface head coil concerning the animal's head to avoid subsequent inaccurate brain extraction and segmentation. The diffusion protocol was set up as a single shell EPI sequence (voxel size = 0.115 × 0.115 × 0.66 mm^3^, with 30 isotropically distributed diffusion-weighted directions, diffusion weightings of 900 s/mm^2^, 5 non-diffusion weighted b = 0 s/mm^2^ images). Very precise positioning of the slice package is requested to prevent any misalignment along the head-tail axis that might introduce strong partial volume effects. FAIR-ASL is characterized by two T1 inversion recovery maps under both global and selective inversion regimes (voxel size = 0.156 × 0.208 × 1 mm^3^, T1 map with 15 inversion times). Measures of endogenous Glutamate levels were performed using a Glu-CEST acquisition with voxel size = 0.2 × 0.2 × 1 mm^3^, with the Z-spectrum generated with 22 off-resonance saturation pulses with lengths = 1 s and intensity = 5 mT.

The harmonized protocol and analysis pipelines allow obtaining the following qMRI measures: the whole brain volumes for 3D T2w, the white matter integrity through the fractional anisotropy, radial diffusion value, and axial diffusion values for DWI, the cerebral blood flow for FAIR-ASL, and the endogenous glutamate levels for Glu-CEST.

According to the outcome of discussions within *WP1* regarding the first pathology to tackle, it was decided to study longitudinally (from 4 to 19 months) the transgenic (TG) mouse model APP/PS1. This model is a well-established model of Alzheimer's disease (42, 43).

#### WP4: e-Infrastructures

*RIN–Neuroimaging Network* identified three different web-based platforms developed under previous European and National calls, such as NeuGRID (44), ARIANNAFORIRCCS (45), and Medical Informatic Platform (MIP) (46) to provide complementary tools.

This project echoes the *FAIR guiding principles* of open science developed by the contemporary neuroscience community researchers for enhancing the reusability of research data (36, 47).

NeuGRID (https://neugrid2.eu) is a High-Performance Computing e-infrastructure aiming to collect a large amount of image data paired with computationally intensive data analyses. NeuGRID has been identified by the Re3data initiative as an official research data repository compatible with the FAIR principle. NeuGRID provides the following services to the *RIN–Neuroimaging Network*: (i) persistent storage and monitoring of harmonized acquisitions of phantoms and murine models with the settings of each scanner checked over time; (ii) imaging analysis using publicly available analysis software and artificial intelligence tools (46). Once data transfer from IRCCS hospitals to the central platform is complete, a QC procedure is automatically triggered for the recently uploaded data (i.e., ACR and FUNSTAR phantoms). QC results are exported to a spreadsheet and PNG snapshots, which are archived along with DICOM images for long-term performance reporting and automatically sent to the user as a final report.

MIP (https://ebrains.eu/service/medical-informatics-platform/), which is cross-linked with NeuGRID, is part of a large-scale European initiative, the Human Brain Project and aims at the integration of multiparametric neuroscientific data using advanced machine learning and deep learning algorithms for the investigation of the human brain in pathologies in the future development of this Network (46).

ARIANNAFORIRCCS platform (https://ariannaforirccs.pi.infn.it/), stemmed from the Arianna project (45) is a web-based research environment for the evaluation of advanced harmonized qMRI protocols on healthy participants. ARIANNAFORIRCCS provides the following services: (i) upload of demographics and brain imaging data; (ii) imaging analysis using public available analysis software.

Each of these platforms responds to a specific need of the *RIN–Neuroimaging Network* and the FAIR principles (36, 47). According to them, data are characterized by the following standards:

##### Findability-Persistent Identification and Description With Appropriate Metadata

Datasets within the web-based platforms are matched with metadata, including metadata directly extracted from DICOM image datasets, along with metadata provided by users at the time of upload. This persistent identifier is assigned to both the data and metadata.

##### Accessibility-Sufficient Storage for Human and Machine Access

Data and metadata can be retrieved using several access methods via standard certified protocols (https). Authentication is required to access data.

##### Interoperability-Structuring in a Way That Allows Plain Collaboration With Other Datasets

A structure made up of codified folder and subfolder, enclosing data and metadata, is used to ensure accessible interoperation of the data. Specifically, users specify the type of acquisition and upload a compressed folder containing DICOM files of the MRI sequence and image analyses are triggered in the RIN–Neuroimaging Network platforms.

##### Reuse-Licensed or Accompanied by Terms and Conditions of Use

The data are released with a clear data use agreement within the Network. Community standards and protocols are used to collect, process, and store data and metadata.

## Discussion

In recent years, the considerable technological progress in the field of neuroimaging has increased the reliability, precision, and sensitivity of the MRI acquisitions, contributing to our understanding of the mechanisms of brain aging and pathological alterations, allowing for more accurate diagnosis and prognosis of neurological diseases, as well as the assessment of effects induced by pharmacological and non-pharmacological interventions through the identification of *in vivo* imaging biomarkers. The *RIN–Neuroimaging network* aims to increase the diagnostic and prognostic power of multimodal MRI data through the generation of “big data” of specific imaging biomarkers for neurological diseases collected at different sites through an integrated multidisciplinary and translational approach. Such an overarching aim can only be achieved through sharing clinical, radiological, and scientific knowledge and ongoing support in this collective effort.

Several factors are known to contribute to scanner variability in multisite studies, including-but not limited to-hardware and software scanner characteristics, data acquisition, and analysis (24, 48). Such sources of variability are often of the same order of magnitude as the disease-related variations, thus they could severely affect the diagnostic and prognostic potential of qMRI parameters (20). Inter-site harmonization strategies are needed to evaluate, manage and limit these sources of variability (20–24). Indeed, the definition of a unique gold standard for MRI protocol harmonization within multisite studies remains challenging. Strategies for sharing procedures and knowledge are key pillars to help overcome this challenge. We have presented here a Network of research hospitals whose first project, supported by Ministerial funds, aimed at converging all sorts of different clinical/preclinical and scientific expertise available between sites to promote sharing know-how of advanced imaging techniques and implement rigorous procedures for “big data” collection. The initiative, supported by the Ministry of Health, aims to reshape research in Italy toward a joint goal, integrating the unique clinical and research experience of IRCCSs, through a network of laboratories offering a global and multilevel approach to neuroscience research.

To date, we mainly focused on three key aspects.

The first one concerns *feasibility*. The trade-off between MRI procedures that are easily implementable in clinical routine and the completeness of the data collected is a driver to successful clinical research (6). This aspect was carefully considered during the definition of the guidelines for the acquisition of a minimum standard clinical MRI protocol as well as in the implementation of QC procedures and the advanced qMRI protocol. In particular, according to the state of the art in the field of neuroimaging and considering the characteristics of the subjects who have to undergo MRIs (children and non-cooperative patients and/or with difficulties in maintaining posture), the implementation of the qMRI protocol should aim to have an acquisition time of <1 h.

Furthermore, we consider a widely documented procedure that uses a traveling brain to assess the harmonized protocol *in vivo* in the human, but adapted to the widespread geographical area of our Network. The traveling brain approach is an effective method for controlling for site differences [e.g., (20, 22, 49–53)]. Using only a traveling brain approach for harmonization would require imaging the same participants at all participating sites, but it would also require significant efforts from participants and sites, particularly in a wide and distributed network (50). Additionally, if new sites are added to the Network, these traveling brains should be available to pursue further acquisitions and the effect of increasing age would need to be considered. Compared to previous traveling brain research, to address these needs, we tried to define a range of acceptability for each of the qMRI parameters for each vendor, based on traveling brain acquisitions, as a benchmark to assess consistency across IRCCSs.

A second aspect is the *cross-cutting nature of these advanced imaging methodologies*. Preclinical qMRI protocols have experienced less standardization compared to their clinical counterparts (6, 31, 32). By harmonizing QC approaches and assessing the reliability and reproducibility of quantitative data across preclinical scanners, on the same animal types and models of disease, it is possible to ensure a more accurate and precise transposition of results in the clinical setting.

Finally, a third and crucial aspect regards *empowerment of knowledge*. The multidisciplinary nature of the working teams, which involve professionals with substantially different yet complementary backgrounds, is the key ingredient to enrich and facilitate the standardization, harmonization, and successful sharing of the qMRI protocols. We have brought together clinical, infrastructure, and scientific competencies, such as knowledge of a wide range of physics principles required for advanced sequence implementations as well as image post-processing abilities and machine learning competencies.

Such skills have no boundaries as the working groups created a real synergy between members, through in-person and online regular events; this, in turn, ensured that all collaborators had easy access to all relevant information (17, 25). The work of the *RIN-Neuroimaging Network* is not ended with these SOPs and protocols, but will continue to integrate methods innovation (e.g., acceleration strategies, acquisition of other imaging biomarkers, novel post-processing algorithms).

The impact that the *RIN–Neuroimaging Network* actions provide to the scientific community can be summarized in four key points:

- *Rationalization* of the human and technological resources through constructive synergies;
- *Standardization* of protocols and analysis methods;
- *Optimization* of acquisition systems using shared QC;
- *Sharing* of procedures and data.

By grounding in these concepts, the *RIN–Neuroimaging Network* is now able to guarantee the effective harmonization and sharing of neuroimaging data to maximize the impact of public investment in scientific research and clinical practice.

## Ethics and Dissemination

The Protocol Study was performed under the Declaration of Helsinki (59th General Assembly of the World Medical Association, Seoul, October 2008) and the Medical Research Involving Human Subjects Act (WMO). The procedures involving human participants were reviewed and approved by the Scientific Committee as part of the clinical and research criteria followed by the Neuroradiological Division. All the procedures described were performed in compliance with security, integrity, and privacy. Data protection is relevant due to the nature of the data, the individuals involved, and the purpose of the *RIN–Neuroimaging Network*, whose goal is to analyze and share information among research centers. Therefore, the data are treated following the General Data Protection Regulation (GDPR - 2016/679). No potentially identifiable human images or data is stored. A Publication Policy has been agreed upon by all participating partners. The Network shares the results of individual WPs through dedicated publications in open access peer-reviewed journals and participation in congresses, reaching a large audience of neuroimaging experts.

## Ethics Statement

The studies involving human participants were reviewed and approved by Ethics Committee Lombardy Region IRCCS Institute of Neurology Carlo Besta Foundation Section. The patients/participants provided their written informed consent to participate in this study. The animal study was reviewed and approved by Ufficio Animal Care Unit of IRCCS - Istituto di Ricerche Farmacologiche Mario Negri - Milano, Fondazione IRCCS Istituto Neurologico Carlo Besta - Milano, Ospedale San Raffaele S.r.l. - Milano, I.R.C.C.S. Neuromed-Pozzilli, and Ceinge Biotecnologie Avanzate S.c.a.r.l. - Napoli.

## The RIN–Neuroimaging Network

Francesco Padelli (Fondazione IRCCS Istituto Neurologico Carlo Besta), Francesco Ghielmetti (Fondazione IRCCS Istituto Neurologico Carlo Besta), Jean Paul Medina (Fondazione IRCCS Istituto Neurologico Carlo Besta), Sara Palermo (Fondazione IRCCS Istituto Neurologico Carlo Besta), Mattia Colnaghi (Istituto Auxologico Italiano–IRCCS), Claudia Morelli (Istituto Auxologico Italiano–IRCCS), Maria Camilla Rossi-Espagnet (IRCCS Istituto Ospedale Pediatrico Bambino Gesù), Lorenzo Figà Talamanca (IRCCS Istituto Ospedale Pediatrico Bambino Gesù), Daniela Longo (IRCCS Istituto Ospedale Pediatrico Bambino Gesù), Chiara Carducci (IRCCS Istituto Ospedale Pediatrico Bambino Gesù), Giulia Lucignani (IRCCS Istituto Ospedale Pediatrico Bambino Gesù), Martina Lucignani (IRCCS Istituto Ospedale Pediatrico Bambino Gesù), Francesca Bottino (IRCCS Istituto Ospedale Pediatrico Bambino Gesù), Chiara Parrillo (IRCCS Istituto Ospedale Pediatrico Bambino Gesù), Emanuela Tagliente (IRCCS Istituto Ospedale Pediatrico Bambino Gesù), Claudia Ruvolo (IRCCS Centro Neurolesi Bonino Pulejo), Lilla Bonanno (IRCCS Centro Neurolesi Bonino Pulejo), Domenico Tortora (Ospedale Pediatrico Istituto Giannina Gaslini), Marco Grimaldi (IRCCS Istituto Clinico Humanitas), Maria Luisa Malosio (IRCCS Istituto Clinico Humanitas), Cira Fundaro' (IRCCS Istituti Clinici Scientifici Maugeri), Alessio Moscato (IRCCS Istituti Clinici Scientifici Maugeri), Denis Peruzo (IRCCS E. Medea dell'associazione “La Nostra Famiglia”), Stefano Bastianello (Fondazione IRCSS Istituto Neurologico Naz.le Mondino), Nikolaos Petsas (IRCCS Neuromed), Lorenzo Carnevale (IRCCS Neuromed), Patrizia Pantano (RCCS Neuromed), Fabio Maria Triulzi (Fondazione IRCCS Ca' Granda Osp. Maggiore Policlinico), Giorgio Conte (Fondazione IRCCS Ca' Granda Osp. Maggiore Policlinico), Antonella Iadanza (IRCCS Ospedale San Raffaele), Andrea Falini (IRCCS Ospedale San Raffaele), Giovanni Giulietti (Fondazione IRCCS Santa Lucia), Raffaele Agati (IRCCS Istituto delle Scienze Neurologiche di Bologna), Claudia Testa (IRCCS Istituto delle Scienze Neurologiche di Bologna), Marco Aiello (IRCCS SDN Istituto di Ricerca), Marco Salvatore (IRCCS SDN Istituto di Ricerca), Marta Lancione (Fondazione IRCCS Stella Maris), Mauro Costagli (University of Genova; Fondazione IRCCS Stella Maris), Fabrizio Levrero (IRCCS Ospedale Policlinico San Martino).

## Author Contributions

ANi, SF, CG, MT, ARe, GFo, DA, RL, FT, and MGB contributed conception and design of the study. ANi, SF, CG, MT, ARe, DA, LB, PB, IC, GD, RG, ML, ANa, FP, AP, GS, VM, SM, FB, ARo, LSP, EA, FA, ER, LP, VC, GFe, AC, MB, CT, CC, LR, CA, SG, MGB, and RIN Neuroimaging Network development of clinical neuroimaging recommendations for harmonization. GFo, DA, EM, LP, AC, CC, MGB, and RIN Neuroimaging Network development of preclinical neuroimaging recommendations for harmonization. MT, ARe, LB, PB, SD, and RIN Neuroimaging Network organized the database and infrastructure. ANi, SF, CG, MT, and MGB wrote the first draft of the manuscript. ARe, GFo, DA, LB, PB, GD, EM, ANa, FP, and GS wrote sections of the manuscript. All authors and RIN Neuroimaging Network contributed to manuscript revision, read, and approved the submitted version.

## Funding

This project was funded by the Italian Minister of Health (RRC-2016-2361095, RRC-2017-2364915, RRC-2018-2365796, and RCR-2019-23669119_001, and RCR 2020-23670067) and the Ministry of Economy and Finance (CCR-2017-23669078).

## Conflict of Interest

The authors declare that the research was conducted in the absence of any commercial or financial relationships that could be construed as a potential conflict of interest.

## Publisher's Note

All claims expressed in this article are solely those of the authors and do not necessarily represent those of their affiliated organizations, or those of the publisher, the editors and the reviewers. Any product that may be evaluated in this article, or claim that may be made by its manufacturer, is not guaranteed or endorsed by the publisher.
