# Supplementary material for: Quantitative MRI Harmonization to Maximize Clinical Impact: The RIN–Neuroimaging Network
Source: Front Neurol. 2022 Apr 14;13:855125. doi: 10.3389/fneur.2022.855125 (PMC9047871; doi:10.3389/fneur.2022.855125)
Supplement: Supplementary file 1 [file Data_Sheet_1.PDF]

ACR Manual

Version 6: 12/13/2021

Contacts: reteimaging@istituto-besta.it

The document was drafted by the WP2 working group

Coordinators: Claudia Gandini Wheeler-Kingshott, Michela Tosetti

Authors: Anna Nigri, Fulvia Palesi, Domenico Aquino, Laura Biagi, Ruben Gianeri, Alice Pirastru

Summary

1. PHANTOM SET-UP & MAINTENANCE 3

1.1 Bubble Removal 3

2. PHANTOM POSITIONING and CENTERING 4

2.1 General recommendations 4

2.2 Phantom ositioning 4

3. ACQUISITION PROTOCOL 7

4. RECOMMENDATION FOR ACQUISITION 10

4.1 Verification of the phantom positioning along the axis of the magnet 10

4.2 Changes to the structure of ACR phantoms 11

5. ACR TESTS 12

5.1 ACR Large 13

5.2 ACR Small 14

6. DATA EXPORT FROM THE SCANNER 15

7. DATA UPLOAD TO THE DATABASE 15

8. REPORT 15

9. LINK 16

1. PHANTOM SET-UP & MAINTENANCE

Small bubbles are normally present in the phantom, even in newly purchased ones. Only in the case of very large bubbles, it is necessary to proceed with the following procedure (see 1.1). In general, distilled water is the evaporating component of the solution, so when bubbles are created it is first necessary to migrate them towards the upper end of the phantom.

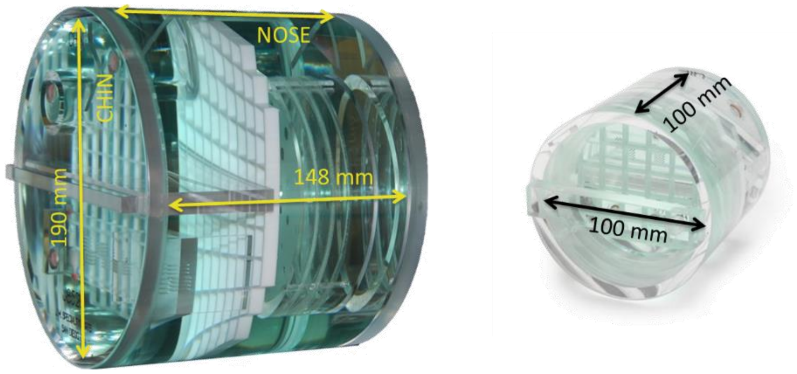

ACR Large *ACR Small*

Figure 1

1.1 Bubble Removal

This procedure is only necessary when the bubbles present in the phantom are larger than 2 cm in diameter. The video at the link below shows how to combine the various bubbles dispersed in the phantom into a single big bubble and how to subsequently fill it with distilled water.

https://youtu.be/1-q8sfI4YJY

As reported in the ACR manual:

Occasionally air bubbles will appear as the phantom ages. When the air bubbles are significant enough to affect the diameter measurement it is time to add fluid (top-off). Do not over-tighten the plugs; they are fragile and prone to breakage.

The solution in the phantom is considered toxic. Do not ingest fluid, or allow it to contact bare skin or eyes. Flush immediately with water in case of contact.

Adding fluid to your phantom:

1) *Only add pure distilled water.*
2) *Prop the phantom with the air bubble positioned under one of the ports.*
3) *Clean the area around the fill port with water, not alcohol. Remove any dust, dirt or debris.*
4) *Use a clean 8mm or 5/16 "socket only (no extra leverage) to gently remove the plug, noting the degree of tightness.*
5) *Using care not to introduce debris into the phantom, add the distilled water with a syringe.*
6) *Moisten a cotton tip applicator and clean around the "O" ring on the plug and sealing area.*
7) *Tighten the plug to the same degree as noted when it was removed. Do not over-tighten, as the plug will break easily.*

2. PHANTOM POSITIONING and CENTERING

2.1 General recommendations

It is recommended to acquire images when the gradients have already been used, typically after the first acquisitions of the day.

Keep the phantom at a temperature between 15-27 °.

2.2 Phantom positioning

It is important that the phantom is positioned as follows.

ACR Large

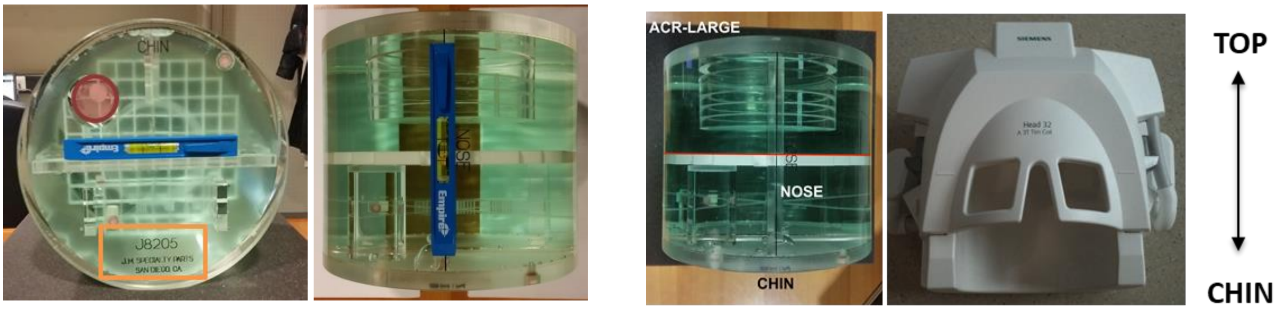

A B C D

ACR Small

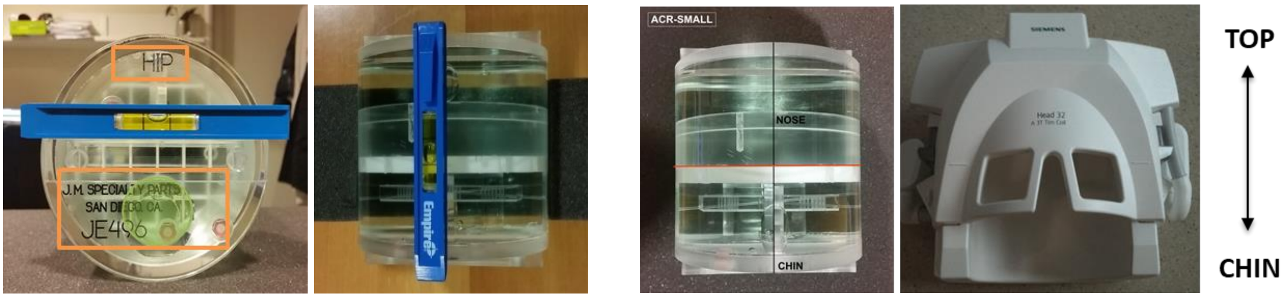

E F G H

Figure 2. Positioning of the phantom

It is recommended that the phantom is placed according to these guidelines:

1. Support for the **ACR large** phantom: the use of a small foam pad - typically the one used for patients - is suggested to avoid direct contact with the coil; it is recommended to keep the same distance from the sides of the coil;
2. Support for the **ACR small** phantom: use the concentric protective discs supplied with the phantom to position it inside the coil. In order to facilitate laser centering, the protective discs could be cut in half (Figure 3); it is recommended to keep the same distance from the sides of the coil;
3. In figure 2A, 2E, the **orange boxes indicate the label for the lower part of the phantom**; label must be positioned (as patient's feet) so that it is readable and is not upside down, as shown in figure 2;
4. the phantom must be **aligned both in the axial and sagittal plane**. For this, you must use the level supplied with the phantom (Figure 2A, 2B, 2E, 2F).

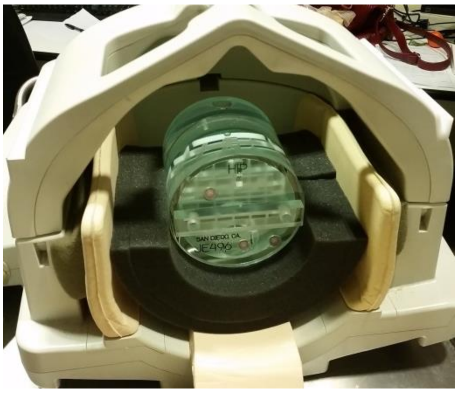

Figure 3. Detail of the ACR small phantom holder

Phantom centering:

1. when the laser is lined up with the top edge (head direction) of the white grid structure, then the phantom is appropriately positioned (as reported in Figure 2C, 2G; Figure 4),

Details for ACR large are given in the ACR recommendations:

https://www.acr.org/-/media/ACR/Files/Clinical-Resources/QC-Manuals/MR_QCManual.pdf

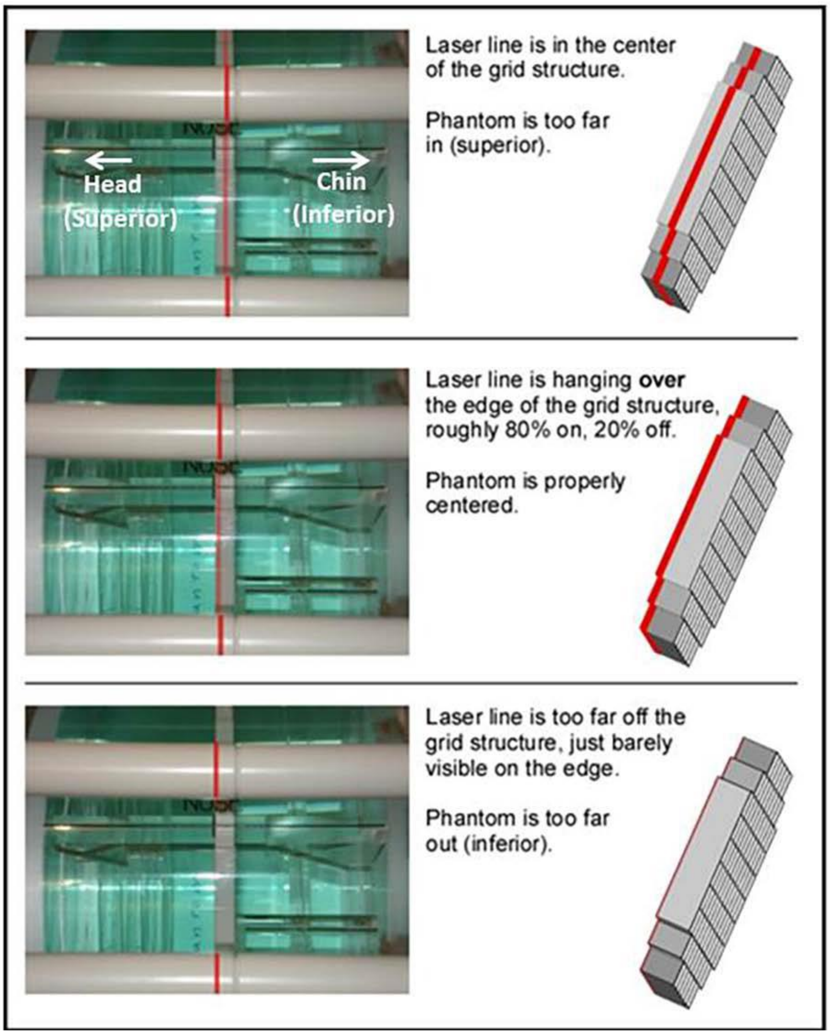

Figure 4. The central box of the figure shows how the phantom centering (both ACR Large and Small) must be carried out using the laser.

3. ACQUISITION PROTOCOL

ACR large and ACR small protocol includes:

1. ***sagittal localizer scan***
2. ***T1 spin-echo sequence***: disable all filters and post-processing applied by the scanner except PURE (GE), CLEAR (Philips), and Prescan Normalize (Siemens) uniformity correction which must be "ON".
3. ***T2 spin-echo sequence (Note 1)***: disable all filters and post-processing applied by the scanner except PURE (GE), CLEAR (Philips) and Prescan Normalize (Siemens) uniformity correction which must be "ON".

Sequences T1 and T2 must have exactly the same spatial parameters.

NOTE 1: perform a single echo sequence. For scanners that do not support a single echo sequence with these parameters (for example Philips), use a double echo sequence with TE1 = 20 ms and TE2 = 80 ms.

The parameters are shown in the following tables:

ACR Large

ACR LARGE	Pulse sequence	TR (ms)	TE (ms)	Orientation	FOV (mm)	Number of slices	Slice thickness (mm)	Slice gap (mm)	Averages	Matrix	Receiver Bandwidth	Filter / options	Scan time (estimate)
Localizer scan	Spin echo	200	20	sagittal	256	1	20	-	1	256x256	-		0:56
T1w ACR	Spin echo	500	20	axial	256	11	5	5	1	256x256	250 ± 20 Hz / px (*)	All disabled, except PURE (GE), CLEAR (Philips), Prescan Normalize (Siemens)	2:16
T2w ACR	Spin echo	2000	80	axial	256	11	5	5	1	256x256	250 ± 20 Hz / px (*)	All disabled, except PURE (GE), CLEAR (Philips), Prescan Normalize (Siemens)	8:56

(*) Receiver Bandwidth: for Siemens 250 ± 20 Hz / px; for Philips set the water-fat shift (WFS) parameter to 1.735 px (range 1.61-1.89 px); for GE use the value ± 31.25 kHz (or as close as possible).

ACR Small

ACR SMALL	Pulse sequence	TR (ms)	TE (ms)	Orientation	FOV (mm)	Number of slices	Slice thickness (mm)	Slice gap (mm)	Averages	Matrix	Receiver Bandwidth	Filter / options	Scan time (estimate)
Localizer scan	Spin echo	200	20	sagittal	120	1	20	-	1	192x192	-		0:32
T1w ACR	Spin echo	500	20	axial	120	7	5	3	1	192x192	250 ± 20 Hz / px (*)	All disabled, except PURE (GE), CLEAR (Philips), Prescan Normalize (Siemens)	1:16
T2w ACR	Spin echo	2000	80	axial	120	7	5	3	1	192x192	250 ± 20 Hz / px (*)	All disabled, except PURE (GE), CLEAR (Philips), Prescan Normalize (Siemens)	5:04

(*) Receiver Bandwidth: for Siemens 250 ± 20 Hz / px; for Philips set the water-fat shift (WFS) parameter to 1.735 px (range 1.61-1.89 px); for GE use ± 25 kHz (or as close as possible).

4. RECOMMENDATION FOR ACQUISITION

Place the acquisition package on the sagittal localizer as shown in the figures below.

In **ACR Large**, verify that slices 1 and 11 correspond to the intersection of wedges in the phantom, as indicated by the yellow circles in Figure 5.

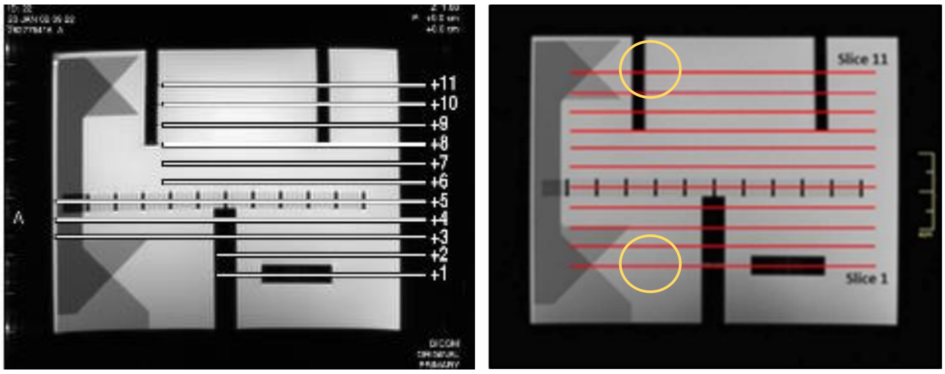

Figure 5. ACR Large (modified from LargePhantomGuidance.pdf); see section 4.2

In **ACR Small**, verify that slice 1 is at the intersection of the wedge forming in the phantom, as indicated by the yellow circle in Figure 6.

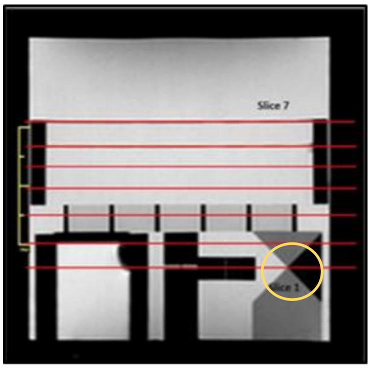

Figure 6. ACR Small (edited by SmallPhantomGuidance.pdf)

4.1 Verification of the phantom positioning along the axis of the magnet

Check the images acquired in T1w to ensure the magnet's z-axis has been correctly aligned: the length of the bars in the first slice should be of similar length, as shown in Figure 7. Figure 8 shows incorrect positioning.

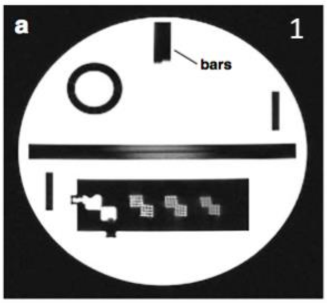

Figure 7. Correct placement (modified from LargePhantomGuidance.pdf)

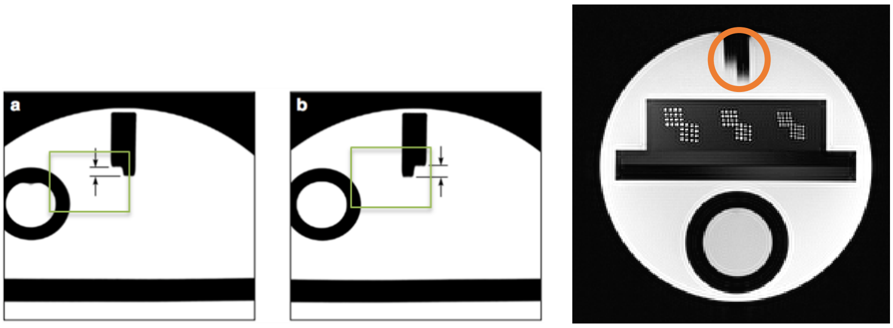

Figure 8. Incorrect positioning (on the left ACR Large, on the right ACR Small), modified by LargePhantomGuidance.pdf

4.2 Changes to the structure of ACR phantoms

ACR large

Slice 5 of the ACR large phantom has been modified by the Manufacturing Company since June 2019 as follows:

https://www.acraccreditation.org/-/media/ACRAccreditation/Documents/MRI/Slice-5-Guidance.pdf

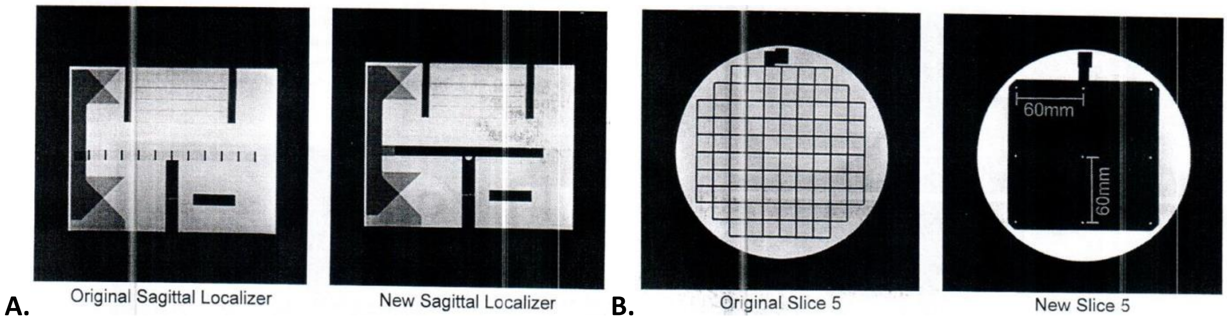

Figure 9. ACR large: on the left (A) Localizer pre and post change; on the right (B) slice 5 pre and post change

ACR small

Slice 4 of the ACR large phantom has been modified by the Manufacturing Company since January 2021 as follows:

https://www.acraccreditation.org/-/media/ACRAccreditation/Documents/MRI/New-Small-Phantom-Slice-4-Grid-Explanation.pdf

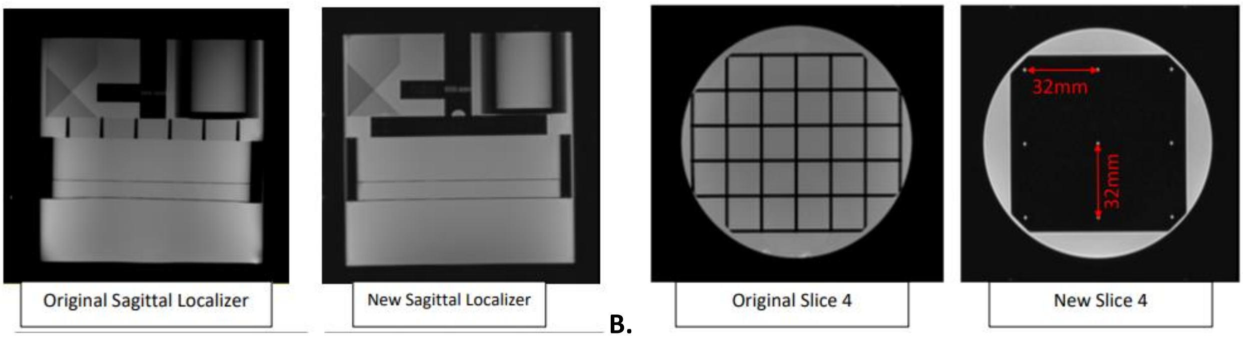

Figure 10. ACR small: on the left (A) Localizer pre and post change; on the right (B) slice 4 pre and post change

5. ACR TESTS

This section includes the tests that were performed on large and small ACR phantom images. More details on each test can be found at this link in LargePhantomGuidance and SmallPhantomGuidance: [https: // www.acraccreditation.org/modalities/mri](https://www.acraccreditation.org/modalities/mri)

The tests carried out are the following: analysis of

- signal uniformity
- ghosting
- geometrical accuracy (including ellipse's ratio)
- slice thickness
- high contrast

The analysis script has been automated and optimized for ACR Large and Small based on the Matlab semi-automatic quality assurance script (http://jidisun.wixsite.com/osaqa-project/resources).

5.1 ACR Large

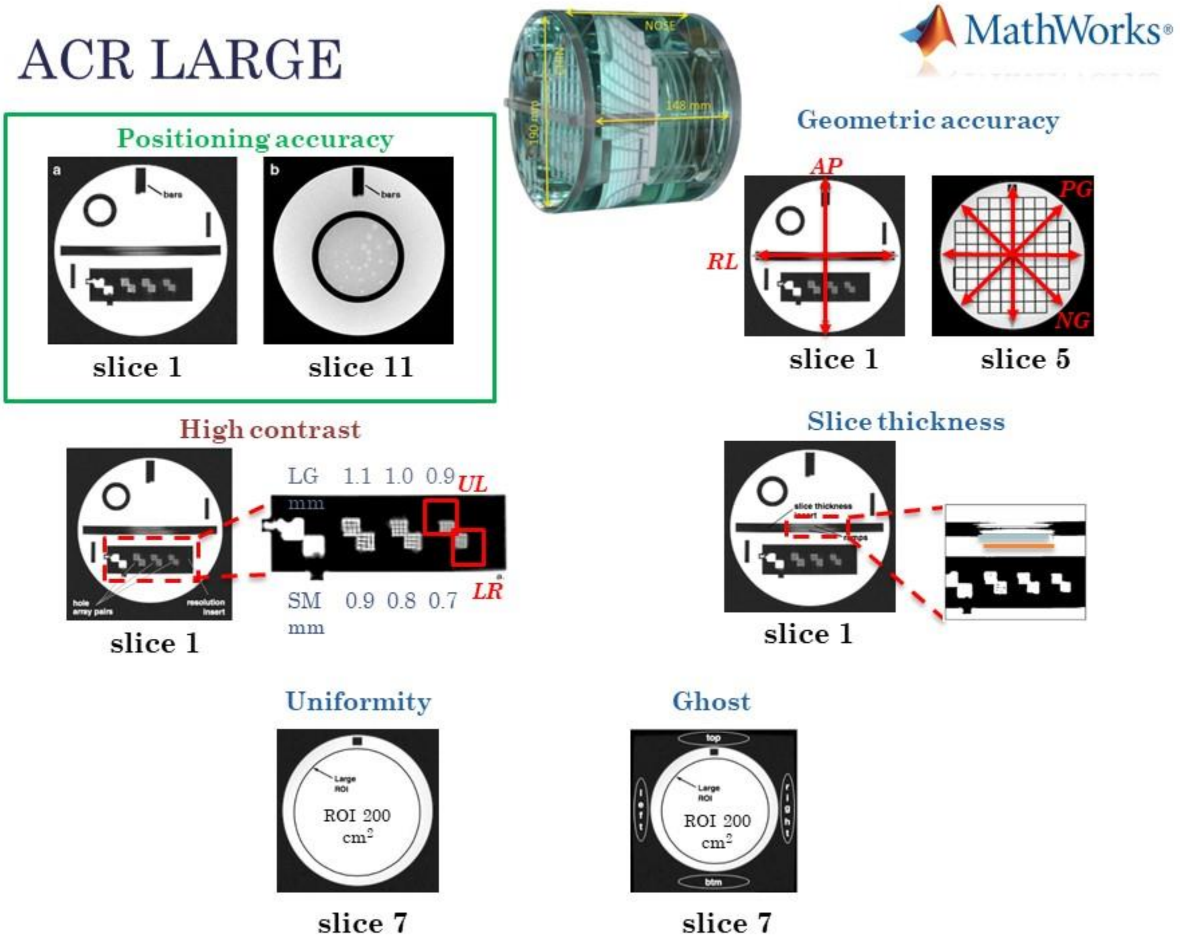

Figure 11. Section on which each test is carried out for the evaluation of geometric and contrast distortions, on ACR large (modified from LargePhantomGuidance.pdf)

5.2 ACR Small

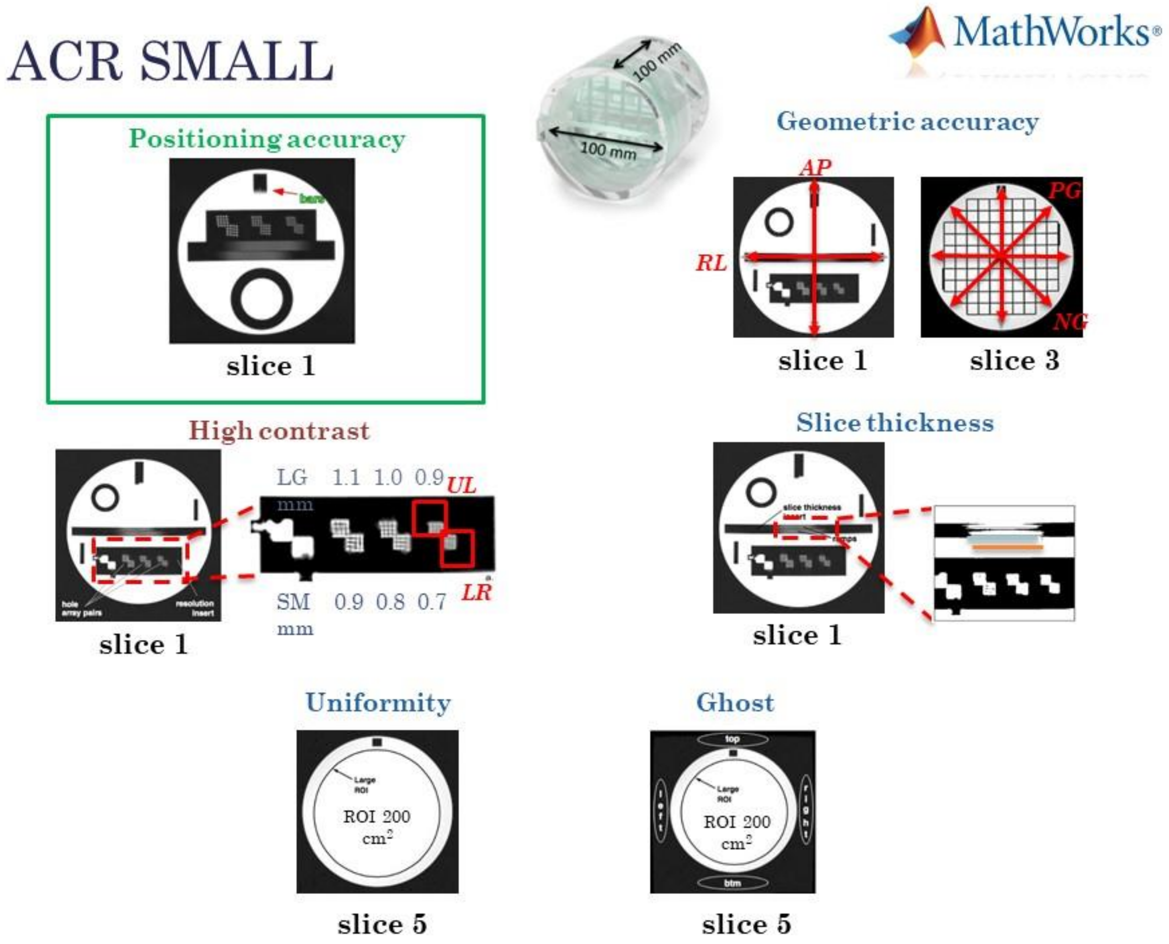

Figure 12. Section on which each test is carried out for the evaluation of geometric and contrast distortions, on ACR small (modified by SmallPhantomGuidance.pdf)

Legend

RL = right-left;
AP = anterior-posterior;
NG = negative gradient;
PG = positive gradient;
UL = upper-left;
LR = lower-right

6. DATA EXPORT FROM THE SCANNER

The data must be exported in non-enhanced DICOM format (not a single DICOM file per sequence).

7. DATA UPLOAD TO THE DATABASE

Upload the data as .zip files thus renaming on neuGRID (http://neugrid2.eu/) as follows:

ACR_L SiteXX_TestXX.zip or **ACR_S SiteXX_TestXX.zip**

where *SiteXX* corresponds to the number of the site (e.g. Site01), L or S corresponds to ACR large or ACR small, and TestXX corresponds to the number of phantom acquisition (e.g. Test01 is the first acquisition, subsequent acquisitions will be Test02, Test03, etc...)

The .zip file must contain 3 folders with the DICOMs of the sequences (Note 2):

- A folder with the localizer
- A single folder with the DICOMs of the T1 sequence - with the string "T1" in the folder name -, without other subfolders;
- A single folder with the DICOMs of the T2 sequence (both echoes of the T2 sequence, when required for Philips scanner; see Note 1) - with the string "T2" in the folder name - with no other subfolders.

Eg: **ACR_Site01_Test01.zip**

- ├ Localizer
- ├ T1W_ACR_L
- ├ T2W_ACR_L

where L = Large; S = Small

NOTE 2: each folder must contain only the DICOM files of the sequence. Avoid inserting files such as .XX_ * .PS_ * inside

8. REPORT

The site technician will receive a .zip report via email. The report will contain:

- Large_Results.xls or Small_Results.xls (excel file): the results obtained for each test with the tolerance range
- Screenshot of the slices on which the analysis was performed for each test. In this way it is possible to check the positioning (see Figures 7 and 8), the presence of any bubbles, and the reason for any values out of range for each test.

An error message will be emailed if the uploaded file does not follow the structure outlined in section 7.

9. LINK

https://www.acraccreditation.org/modalities/mri

https://www.acraccreditation.org/-/media/ACRAccreditation/Documents/MRI/LargePhantomGuidance.pdf

https://www.acraccreditation.org/-/media/ACRAccreditation/Documents/MRI/SmallPhantomGuidance.pdf

https://www.acr.org/-/media/ACR/Files/Clinical-Resources/QC-Manuals/MR_QCManual.pdf

https://youtu.be/1-q8sfI4YJY
