# Supplementary material for: Quantitative MRI Harmonization to Maximize Clinical Impact: The RIN–Neuroimaging Network
Source: Front Neurol. 2022 Apr 14;13:855125. doi: 10.3389/fneur.2022.855125 (PMC9047871; doi:10.3389/fneur.2022.855125)
Supplement: Supplementary file 2 [file Data_Sheet_2.PDF]

rin | rete IRCCS delle neuroscienze e della neuroriabilitazione

FUNSTAR Manual

Version 2: 09/09/2019

Contacts: reteimaging@istituto-besta.it; antonio.napolitano@opbg.net

The document was drafted by the WP2 working group

Coordinators: Claudia Gandini Wheeler-Kingshott, Michela Tosetti

Authors: Antonio Napolitano

Summary

1. PHANTOM 3
2. PHANTOM POSITIONING and CENTERING 3
2.1 Phantom positioning 3
2.1 Phantom centering 4
3. ACQUISITION PROTOCOL 4
4. RECOMMENDATION FOR ACQUISITION 6
5. DATA EXPORT FROM THE SCANNER 6
6. DATA UPLOAD TO THE DATABASE 6
7. REPORT 6
8. REFERENCES 7

1. PHANTOM

The FUNSTAR (Functional Stability Reference - Gold Standard) phantom for echo planar imaging (EPI) stability assessment consists of a 17 cm diameter sphere filled with agar gel. The following protocol follows the guidelines defined by Friedman et al., 2006 ('Report on a multicentre fMRI quality assurance protocol').

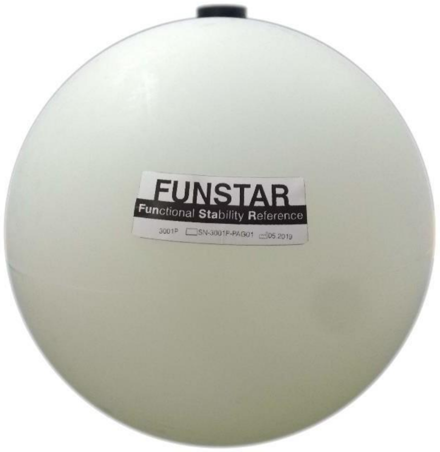

Figure 1

2. PHANTOM POSITIONING and CENTERING

2.1 Phantom positioning

The phantom must be placed as reported in Figure 2.

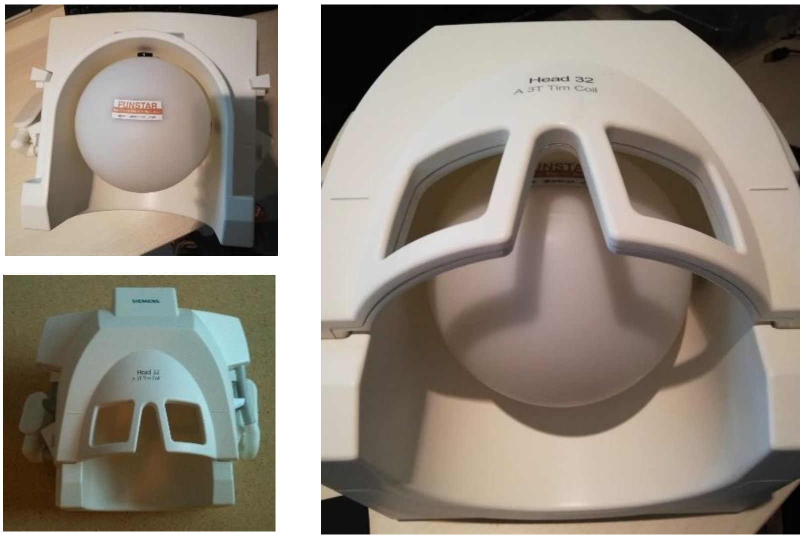

Figure 2. Phantom positioning

In particular, it is required to perform the positioning according to the following guidelines:

- Place the phantom in a lying position; the upper extremity (recognizable by the black element) must face the inside of the scanner (patient head);
- Fix the phantom in position using a small foam pad (the one used for patients);

2.1 Phantom centering

Centering should be carried out according to the following guidelines:

- Locate the laser at the junction line of the hemispheres (raised edge).

3. ACQUISITION PROTOCOL

The FUNSTAR protocol includes:

- **LOCALIZER**: coronal centering in the central plane of the phantom
- **FBIRN_no_shimming**: single volume acquisition, with the shimming option set on standard/tuned-up/first-order
- **FBIRN_con_shimming**: single volume acquisition, with the shimming option set on advanced (PB-volume)
- **FBIRN_first_acquisition**: 200 volumes acquisition, with the shimming option set on advanced

The table with the parameter values for each acquisition is shown below.

Acquisition	TR (ms)	TE (ms)	FA (degrees)	FOV (mm)	# slices	# volumes	Slice thicknes s(mm)	Slice spacin g(mm)	Matrix	Bandwidth (Hz/px)	Slice acquisitio norder	Readout direction	Phase Encoding direction	Shimming	Filter/ options	Scan time
LOCALIZER<br>***(owned by the IRCSS)***																
FBIRN_no_shimming	2000	30	90	220	32	1	4	5	64x64	1595	Interleaved	RL	PA	Standard	All filters disabled<br>NO fat suppression NO parallel imaging	2 sec

Table 1. Acquisition parameters for each sequence

RL: Right-Left

PA: Posterior-Anterior

** It is required to acquire the 'FBIRN_first_acquisition' in both phase-encoding directions (AP and PA)

4. RECOMMENDATION FOR ACQUISITION

Place the acquisition package so that it surrounds the entire phantom, as shown in Figure 3.

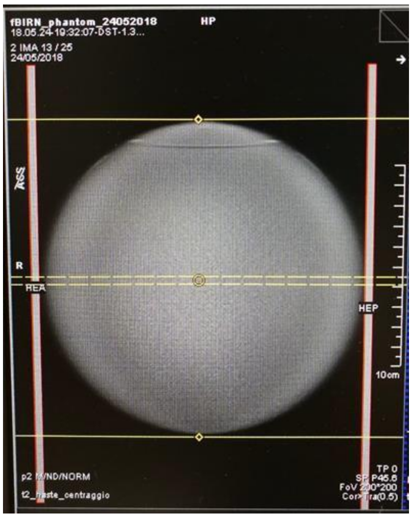

Figure 5. FUNSTAR phantom acquisition

5. DATA EXPORT FROM THE SCANNER

For the analysis, data must be exported in DICOM format.

NB: when using a Philips scanner, export data in non-enhanced DICOM format.

6. DATA UPLOAD TO THE DATABASE

Upload data in .zip format to neuGRID (http://neugrid2.eu), renaming the folder as shown below:

FUNSATR_ SiteXX_dd_mm_yyyy.zip

This zipped folder must contain the following subfolders for each DICOM sequence:

- SiteXX _dd_mm_yyyy_FBIRN_localizer
- SiteXX_dd_mm_yyyy_FBIRN_con_shimming
- SiteXX _dd_mm_yyyy_FBIRN_no_shimming
- SiteXX_dd_mm_yyyy_FBIRN_acquisition

7. REPORT

The site technician will receive a .zip report via email. The report will contain:

- FUNSTAR_Results.pdf (excel file): the results obtained for each test with the tolerance range

An error message will be emailed if the uploaded file does not follow the structure outlined in section 6.

8. REFERENCES

Friedman L, Glover GH. Report on a multicenter fMRI quality assurance protocol. J Magn Reson Imaging. 2006 Jun;23(6):827-39. doi: 10.1002/jmri.20583. PMID: 16649196.
